# An implementation study of electronic assessment of patient-reported outcomes in inpatient radiation oncology

**DOI:** 10.1186/s41687-022-00478-3

**Published:** 2022-07-19

**Authors:** Thomas Nordhausen, Katharina Lampe, Dirk Vordermark, Bernhard Holzner, Haifa-Kathrin Al-Ali, Gabriele Meyer, Heike Schmidt

**Affiliations:** 1 Institute of Health and Nursing Science, Medical Faculty, Martin Luther University Halle-Wittenberg, Halle (Saale), Germany; 2 Department of Radiation Oncology, University Hospital Halle (Saale), Martin Luther University Halle-Wittenberg, Halle (Saale), Germany; 3 University Hospital of Psychiatry II, University Hospital Innsbruck, Innsbruck, Austria; 4 Evaluation Software Development, Innsbruck, Austria; 5 Department of Internal Medicine IV, University Hospital Halle (Saale), Halle (Saale), Germany; 6 Krukenberg Cancer Center, University Hospital Halle (Saale), Halle (Saale), Germany

**Keywords:** Patient-reported outcomes, Electronic assessment, Implementation, Health-related quality of life, Radio-oncology

## Abstract

**Purpose:**

Despite evidence for clinical benefits, recommendations in guidelines, and options for electronic data collection, routine assessment of patient-reported outcomes (PROs) is mostly not implemented in clinical practice. This study aimed to plan, conduct and evaluate the implementation of electronic PRO (e-PRO) assessment in the clinical routine of an inpatient radiation oncology clinic.

**Methods:**

The guideline- and evidence-based, stepwise approach of this single-center implementation study comprised preparatory analyses of current practice, selection of assessment instruments and times, development of staff training, and evidence-based recommendations regarding the use of the e-PRO assessment, as well as on-site support of the implementation. Process evaluation focused on potential clinical benefit (number of documented symptoms and supportive measures), feasibility and acceptance (patient contacts resulting in completion/non-completion of the e-PRO assessment, reasons for non-completion, preconditions, facilitators and barriers of implementation), and required resources (duration of patient contacts to explain/support the completion).

**Results:**

Selection of instruments and assessment times resulted in initial assessment at admission (EORTC QLQ-C30, QSR 10), daily symptom monitoring (EORTC single items), and assessment at discharge (EORTC QLQ-C30). Recommendations for PRO-based clinical action and self-management advice for patients concerning nine core symptoms were developed. Staff training comprised group and face-to-face meetings and an additional e-learning course was developed. Analyses of clinical records showed that e-PRO assessment identified more symptoms followed by a higher number of supportive measures compared to records of patients without e-PRO assessment. Analysis of n = 1597 patient contacts resulted in n = 1355 (84.9%) completed e-PROs (initial assessment: n = 355, monitoring: n = 967, final assessment: n = 44) and n = 242 (15.2%) non-completions. Instructions or support to complete e-PROs took on average 5.5 ± 5.3 min per patient contact. The most challenging issue was the integration of the results in clinical practice.

**Conclusion:**

E-PRO assessment in oncologic inpatient settings is acceptable for patients and can support symptom identification and the initiation of supportive measures. The challenge of making the “data actionable” within the clinical workflow and motivating clinical staff to use the results became evident.

**Supplementary Information:**

The online version contains supplementary material available at 10.1186/s41687-022-00478-3.

## Plain English summary

Cancer patients’ perceptions regarding their symptoms and functioning are important as they can differ from a professional assessment. Patients’ perceptions and self-assessment can be collected via electronic devices. Thus, the clinical staff can see a graphic overview of individual disease-related burden. Despite studies indicating the benefit of this assessment for care and symptom management, it is not integrated into routine care so far. The aim of our study was, to plan, conduct and evaluate the implementation of electronic patient-reported assessment in a radio-oncology inpatient clinic under “real-life” clinical conditions instead of study conditions. Patients could complete an electronic assessment at the beginning/end and during their treatment. Results indicate that electronic self-assessment can identify more symptoms than the assessment of physicians and nurses. Patients completing a self-assessment are more likely to receive supportive measures. The majority of 80–90% of patients were willing to complete a self-assessment. On average 5–6 min were needed to explain or support the completion. While the intervention was feasible and acceptable for patients, motivating clinical staff using its results was most challenging. The importance of technical support became evident.

**Supplementary Information:**

The online version contains supplementary material available at 10.1186/s41687-022-00478-3.

## Background

Patients’ perceptions of cancer or therapy-related burden are subjective and can differ from healthcare professionals (HCPs) perceptions [1, 2]. Therefore, routine assessment of patient-reported outcomes (PROs) is recommended to systematically capture individual perceptions of health conditions directly from patients, such as health-related quality of life (HRQOL) including functionality and symptoms [3, 4]. Studies have shown that PROs complementing routine assessment are a feasible intervention and acceptable for patients as well as HCPs. They can support communication and the integration of patient perspectives in clinical decision-making, treatment, and care, thus improving patient satisfaction and symptom management [5–8]. In a large randomized controlled trial, PRO assessment was associated with fewer unplanned hospital admissions and increased survival [9]. International guidelines and organizations for cancer care recommend the integration of PROs into clinical practice [10–13]. Electronic data capture with mobile devices can facilitate clinical implementation due to variable modes of access, computerized evaluation, and graphic display of results [14].

Despite this evidence, systematic PRO assessment is still not integrated into most oncological settings [15, 16], indicating that there is a large difference between studies favoring its feasibility and acceptability and actual clinical practice. Most studies focus on evaluating PROs within study conditions [17], including the recruitment of predefined participant groups for a certain period of time supported by additional personal and time resources. While study conditions are useful for developing and pretesting PRO-based interventions, they may reflect clinical practice not sufficiently enough to gain adequate knowledge regarding long-term integration in “real-life” application contexts. The implementation of systematic PRO assessment into practice is complex and challenging because of the number of stakeholders involved, organizational and financial aspects, and the need to change clinical routines [10]. Major barriers are the reluctance of organizations and HCPs to change established practice due to reservations regarding PROs, the lack of resources (time, staff, finances), and data protection or IT issues [18–21]. Existing guidelines provide general recommendations and advice for the implementation of PRO assessment into clinical practice [10, 11, 22]. However, when aiming for a successful implementation process, specific facilitators and barriers for each setting, current clinical practice, attitudes of all relevant stakeholders (e.g. presumptions and concerns), and the potential benefit of PROs should be explored and addressed [20, 23, 24]. Targeted training improving knowledge and competencies can facilitate the clinical use of PRO assessment [10, 25, 26]. Following these recommendations and guidelines this study aims to plan, conduct and evaluate the implementation of electronic PRO (e-PRO) assessment in inpatient clinical oncology. Addressing a lack of implementation studies regarding long-term applications in the clinical routine [17], our approach focuses on gaining comprehensive and quantifiable insight into the experiences of the implementation process under “real-life” clinical conditions by using a reproducible descriptive method of evaluation. Our methods and results can be used as a basis to further develop and adapt implementation and evaluation strategies in other oncology settings. The following research questions are addressed:

- Which necessary preconditions, barriers, and facilitators can be identified?
- Which are the potential benefits of e-PRO assessment regarding symptom management?
- How feasible and acceptable is e-PRO assessment?
- Which resources are needed for an e-PRO assessment?

## Methods

### Study design and setting

Aiming to examine preconditions and feasibility and to gain experiences and insights for the planning of broader implementation in other departments of the Krukenberg Cancer Center, University Hospital Halle (Saale), a single-center design was chosen. Because of the heterogeneity of diagnoses and treatments which include both chemotherapy and radiotherapy representing a broad spectrum of cancer treatment and care, the inpatient clinic of the radiation oncology department, comprising 34 beds, was chosen for this implementation.

The majority of radiotherapy in Germany is delivered in an outpatient setting. The reasons for inpatient radiotherapy are predominantly the delivery of concomitant chemoradiation (in particular the days of chemotherapy delivery, during a series of radiotherapy) and poor general condition with the need for intensive supportive therapy, e. g. intravenous medication or palliative therapy [27].

### Stepwise approach

Due to the complexity of the implementation process, the Medical Research Council (MRC) framework for the development and evaluation of complex interventions [28] guided the approach of this study, containing the three steps development, implementation and evaluation. In addition, to model the procedure specifically for e-PRO assessment, the Manual of the European Organisation of Research and Treatment of Cancer (EORTC) for the use of EORTC measures in clinical practice [10] and evidence regarding relevant aspects like facilitators and barriers of PRO assessment were taken into account [5, 20, 23, 24].

The three steps and the procedure within are described following.

### Development

Aiming to provide information on current practice, a purposive sample of n = 15 patient records comprising various cancer diagnoses and treatments was analyzed focusing on routinely documented PROs. Participating observation of the clinical practice of physicians and nurses comprised protocols of routine processes and clinical workflow, potential facilitators, barriers, and further aspects relevant for implementation. A self-developed, semi-structured survey of HCPs working in the radiation oncology department was conducted to explore their knowledge and views on e-PRO assessment. The survey consisted of six items regarding the understanding of HRQOL and attitudes about the potential of e-PRO assessment for supporting cancer care and personal practice, possible effects on the personal workload, usability, and acceptance or burden for patients. Each item could be answered via bipolar Likert scale (Yes, rather yes, rather no, no) with an additional, seperate option for “don’t know” and a free-text field.

Aiming to tailor the set-up of e-PRO assessment to the setting of radiation oncology, a multi-professional focus group with health care professionals (HCPs, including physicians, nurses, psycho-oncology) from the department was conducted to discuss the clinical procedure, decide on assessment instruments and times, and the requirements for the assessment software [23]. Minutes were taken and the decisions consented.

Interdisciplinary group-based training sessions for HCPs, focusing on the use of e-PRO assessment in clinical practice and the discussion of implementation issues and concerns, were developed and conducted, complemented by on-site face-to-face training regarding the technical use. To ensure sustainability and facilitate training of new HCPs, an e-learning course was developed, adapted, and finalized throughout the implementation process allowing the individual choice of timing and content according to the learning needs of the users.

In addition, after first experiences with the e-PRO assessment, the desire for easily accessible and structured guidance on how to react to reported symptoms was expressed. To meet this need, clinical recommendations for relevant symptoms were developed aiming to guide clinical consequences of e-PRO results [29].

### Implementation

According to the study aims of implementation of e-PRO assessment in clinical routine including assessment of potential benefit, feasibility and barriers under routine conditions, in accordance with a declaration of no-objection by the ethics committee of the Medical Faculty, Martin-Luther-University Halle-Wittenberg (declaration from 08.01.2019, retrospectively assigned process number: 2022-061), no informed consent of patients was obtained to avoid a possible selection bias. Thus, the collection of e-PROs could be offered to all inpatients of the radio-oncology clinic except patients with severe cognitive impairment. All patient contacts to explain and/or support the completion of the e-PRO assessments were conducted by the study staff.

The implementation process consisted of the following three phases:

- The initial phase focused on technical adjustments to achieve full functionality of the assessment software in connection with the clinical information system and electronic patient records.
- The consolidation phase focused on optimized integration of e-PRO assessment into clinical routine and requirements for further adaptation to enhance clinical use of the e-PRO assessments and patients’ self-assessments.
- The routine phase focused on constant data flow and analyses of necessary resources aiming to further facilitate the integration into clinical practice.

The implementation was accompanied by on-site monitoring in all three phases, resulting in process adaptation and optimization. Emerging issues were addressed in team meetings.

### Evaluation

The software CHES (Computer-based Health Evaluation System) [30] was used to collect e-PROs, including scoring, compilation, and graphic presentation of the results within the clinical records.

Data collection for the evaluation focused on potential clinical benefit, feasibility/acceptance, and required resources. Potential clinical benefit was operationalized through comparison of the number of recorded symptoms (e-PROs vs. documentation of physicians and nurses) and initiated supportive measures (patients without vs. patients’ completion of e-PROs) for three samples of n = 100 each:

- Sample 1: Without the opportunity for e-PRO assessment.
- Sample 2: With e-PRO assessment, without integration of results into clinical records.
- Sample 3: With e-PRO assessment, with the integration of results into clinical records.

These samples were extracted randomly through the automatized allocation of numbers for e-PRO assessment at therapy start (IA1, IA2, and IA3) and during the course of therapy (SM1, SM2, and SM3). The e-PRO results as well as sociodemographic and medical characteristics were extracted automatically from CHES, recorded symptoms and initiated supportive measures were extracted manually from the clinical documentation based on defined assignment criteria.

Furthermore, all patient contacts from the beginning of the consolidation phase were documented via standardized protocols by the study staff. Feasibility and acceptance were operationalized through the percentage of contacts resulting in completion or non-completion of the e-PRO assessment, characteristics of patients completing/not completing, and reasons for non-completion. Required resources were operationalized through the time required for patient contacts to explain and/or support the completion. Due to ethical requirements, these protocols comprised only anonymized data.

Notes were taken to document on-site monitoring, face-to-face coaching, and team meetings with a focus on relevant issues, e.g. facilitators or occurring barriers during the implementation process.

For data analysis, quantitative data were extracted or transferred into SPSS Version 25 and analyzed descriptively. Descriptive statistics covered frequency, mean-values, and standard deviation, minimum and maximum. All data were anonymized and aggregated for analysis and reporting. Notes were summarized narratively.

Since the study was not designed to examine or prove hypotheses, no inferential statistical analyses were performed.

## Results

### Analysis of current practice

The review of clinical records revealed a routine assessment of emotional burden (QSC-R10 [31]), nutrition (NRS [32]), and dichotomous questions for dyspnea, insomnia, constipation, and diarrhea at admission. Pain was assessed three times a day via a numeric rating scale (0–10). Documentation of other symptoms was not standardized.

Participating observation led to the identification of the following facilitators for the implementation: regular admission times and existing technical equipment with electronic clinical records, individual touchscreens next to each bed, and computer-supported ward rounds. Potential barriers comprised concerns of HCPs (e.g. doubts regarding patients’ ability to complete e-pro assessments) and time constraints due to temporary staff shortages.

The survey was completed by 20 out of 30 HCPs (67%), i.e. n = 12 (60%) physicians, n = 7 (35%) nurses and n = 1 (5%) case manager. All participants reported knowledge about the concept of PROs (n = 20, 100%), and the majority considered e-PRO assessment a contribution for improving cancer care (n = 18, 90%) or supporting their own work (n = 19, 95%). Twelve (60%) expected increased workload due to e-PRO assessment, n = 10 (50%) doubted or were unsure regarding easy use by patients, and n = 8 (40%) considered e-PRO assessment a potential burden for patients.

### Set-up of e-PRO assessment

The focus group decided on an initial assessment of HRQOL at admission with the EORTC QLQ-C30 [33], complemented by the assessment of emotional burden using the QSC-R10 which was already part of the clinical routine as a paper–pencil version. During therapy, daily monitoring of eleven core symptoms with single items from the EORTC Item Library was planned [34]. In addition, specific symptoms of the six most frequent cancer sites treated in the clinic (lung, head-neck, colorectal, brain, breast, prostate) with 3–8 single items were included, allocated automatically based on the individual ICD-10 diagnosis. Before the end of the treatment, the completion of a final HRQOL assessment was planned (EORTC QLQ-C30).

**Fig. 1:**
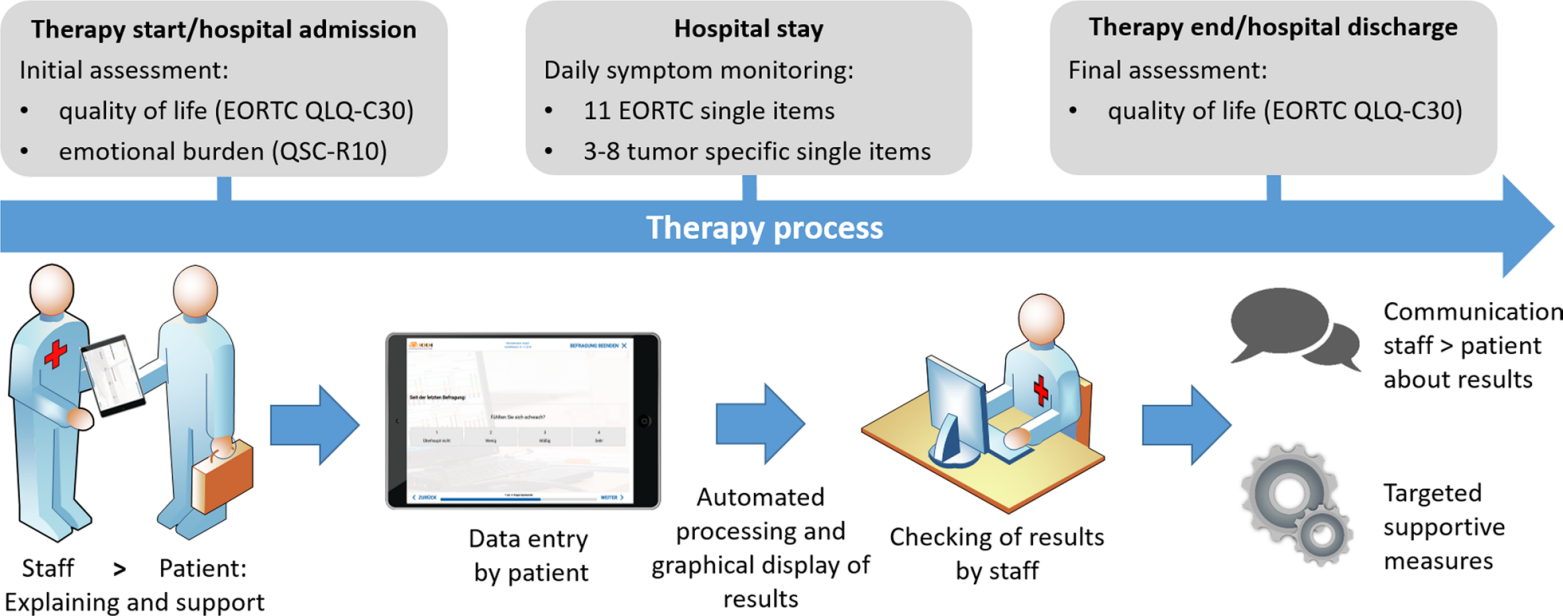
Overview of the assessment instruments and clinical procedure

E-PROs were assessed either via tablets or individual devices located next to each patient’s bed working as an entertainment system. The technical process of the e-PRO assessment is described in Additional file 1. Secure access to the bedside devices was provided through chip cards for individual identification. Data entry via touchscreen and automatic progression to the next question allowed easy use even for patients without prior IT experience (Fig. 1).

**Fig. 2:**
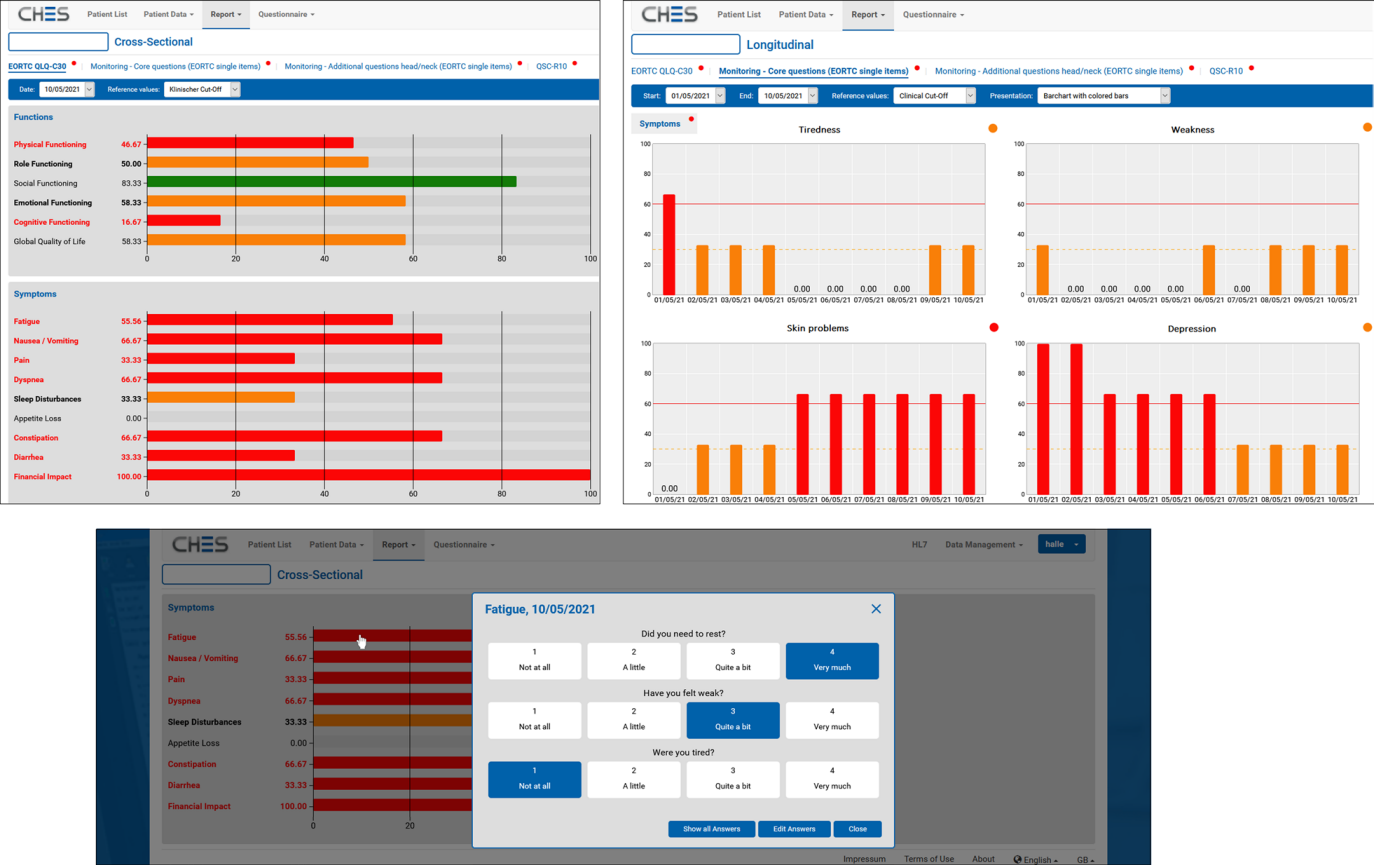
Example for a cross-sectional (top-left) and longitudinal display (top-right) of results including single questions (bottom)

Graphic displays of the e-PRO results were made available in real-time in the electronic patient records including bar charts for a single assessment time (cross-sectional) or all available assessment times for a defined period (longitudinal e.g. monitoring during hospital stay). The color-coded graphic display allows intuitive interpretation of the results (e.g. red = severe burden); the respective scales include a mouseover option to access the scores. Via mouse click all underlying questions are displayed (Fig. 2). The color-coded categorization was based on the thresholds by Giesinger et al. [35] or on equal tripartition in case there were no scientifically based thresholds available for the chosen instrument.

### Training of healthcare professionals

The group and on-site training covered basic knowledge such as the definition of PROs and clinical benefits, the process of e-PRO assessment, technical aspects and use of the system in connection with the electronic patient records, interpretation of results, PRO-based patient-communication, and supportive measures including counseling and self-management recommendations for patients.

The content of the e-learning course was presented mainly through written information and graphics (e.g. pictures, illustrations, flowcharts, partially interactive), complemented by interactive methods and quiz options to assess the learning progress. To demonstrate the integration of e-PROs in clinical situations and communication, videos with HCPs and actor-patients complemented the program.

### Development of clinical recommendations

Guideline-based [36, 37], multi-professional recommendations for HCP were developed for a core set of nine symptoms (fatigue, nausea/vomiting, emotional burden, pain, sleep problems, appetite loss, constipation, diarrhea, skin problems). The recommendations included suggestions for PRO-based communication to gain additional information (e. g. questions to help patients specify their symptoms), further diagnostics (e. g. differential diagnosis), supportive measures, and patient consultations regarding measures of self-management.

In addition, patient self-management recommendations for these symptoms were developed and made available as handouts. These recommendations included suggestions for PRO-based self-observation and general or specific supportive measures patients can conduct independently.

### Implementation

Technical adaptation during the initial phase comprised the optimization of functionality within the clinical IT (e. g., not all inpatients appeared in CHES) and the activation of CHES for the devices next to the patients’ beds. A secure port between CHES and the electronic patient records was created enabling HCPs to access the results without entering an additional password. The QSC-R10 was transferred subsequently into CHES.

During the consolidation phase, the e-PRO assessment results were integrated into the electronic patient records to further simplify their access. To enhance visibility, new results were highlighted in the same way as new medical reports for physicians. Some routine procedures such as ward rounds were accompanied by the first author presenting the results to encourage the use of the results of the assessment.

Within the routine phase, we aimed to facilitate the implementation by generating a constant data flow. Five research assistants were employed to guarantee a daily time slot of one person instructing the patients and documenting the patient contacts.

### Results of the initial assessment and symptom monitoring

During the data collection period between 02/2019 and 02/2021, 568 patients (n = 201 female, n = 367 male) with heterogeneous cancer diagnoses and a mean age of 64.9 years (± 11.6), completed the initial assessment. Comprehensive sociodemographic and clinical characteristics of the sample are presented in Additional file 2, the complete results of the initial assessment with the EORTC QLQ-C30 in Additional file 3.

More than 50% of the patients reported moderate or severe burden on the EORTC QLQ-C30 (scales 0–100, thresholds for severe burden based on Giesinger et al. [35], thresholds for moderate burden on nomal values of EORTC QLQ-C30 scales in the general population [38]) for the following scales: fatigue (n = 451, 79.4%), physical function (n = 412, 72.5%), global quality of life (n = 386, 68.0%), pain (n = 353, 62.2%), insomnia (n = 342, 60.2%), emotional function (n = 312, 54.9%) and dyspnea (n = 296, 52.1%). The QSC-R10 was completed by fewer patients (n = 472) since the electronic assessment was added to the initial assessment during the study. A total of n = 109 (23.1%) stated a high emotional burden ≥ 15 points. From a total of 511 completing patients, n = 106 (20.7%) expressed the need for psycho-oncological support and n = 47 (9.2%) were already receiving support at the beginning of their therapy.

The daily symptom monitoring resulted in 1774 assessment time-points from 344 patients, mean age 63.7 (± 11.0, n = 128 female, n = 216 male). Comprehensive sociodemographic and clinical characteristics of the sample are given in Additional file 4, complete results of the symptom monitoring in Additional file 5.

Patients completed the monitoring questions repeatedly depending on their length of stay and compliance (1–36 times). The symptoms with a moderate or severe burden (scales 0–100, thresholds based on tripartition since there are no scientifically based thresholds for EORTC single items) rated at more than half of all assessment times were tiredness (n = 1306, 73.6%), weakness (n = 1083, 61.1%), and insomnia (n = 989, 55.8%).

### Potential clinical benefit of initial assessment and symptom monitoring

Regarding the initial assessment, the three samples (IA0, IA1, IA2) had similar sociodemographic and clinical characteristics (Additional file 6). In comparison with clinical records, the initial assessment with e-PROs captured more symptoms. The smallest differences were found for symptoms that were already part of the standard documentation (pain, insomnia), the largest for fatigue, appetite loss, and financial difficulties. Results of the comparison of clinical records and the initial assessment regarding the number of identified symptoms are summarized in Table 1.

**Table 1:** Comparison of clinical records and initial assessment regarding the number of identified symptoms (n = 100/sample)

Symptom (day of admission)	Clinical documentation*				Initial assessment (EORTC QLQ-C30)			
	Sample	IA0	IA1	IA2	Sample	IA0	IA1	IA2
Reduced emotional function	Physician	4	10	8	Moderate	–	36	28
	Nurse	6	9	7	Severe	–	24	22
	Total	**8**	**6**	**11**	Total	**–**	**60**	**50**
Fatigue	Physician	5	3	4	Moderate	–	27	27
	Nurse	2	1	1	Severe	–	50	47
	Total	**7**	**3**	**5**	Total	**–**	**77**	**74**
Nausea/vomiting	Physician	5	3	3	Moderate	–	0	0
	Nurse	6	3	2	Severe	–	27	14
	Total	**8**	**4**	**4**	Total	**–**	**27**	**14**
Pain	Physician	26	17	28	Moderate	–	14	8
	Nurse	37	26	40	Severe	–	52	51
	Total	**43**	**30**	**47**	Total	**–**	**66**	**59**
Dyspnea	Physician	5	4	9	Moderate	–	0	0
	Nurse	32	31	28	Severe	–	50	53
	Total	**32**	**31**	**31**	Total	**–**	**50**	**53**
Insomnia	Physician	3	4	4	Moderate	–	28	25
	Nurse	42	36	31	Severe	–	30	28
	Total	**42**	**38**	**34**	Total	**–**	**58**	**53**
Appetite loss	Physician	1	2	2	Moderate	–	24	20
	Nurse	0	2	0	Severe	–	23	21
	Total	**1**	**3**	**2**	Total	**–**	**47**	**41**
Constipation	Physician	7	6	0	Moderate	–	10	10
	Nurse	6	14	5	Severe	–	15	14
	Total	**9**	**16**	**5**	Total	–	**25**	**24**
Diarrhea	Physician	1	2	1	Moderate	–	0	0
	Nurse	1	2	3	Severe	–	19	18
	Total	**2**	**2**	**3**	Total	**–**	**19**	**18**
Financial difficulties	Physician	0	0	0	Moderate	–	0	0
	Nurse	0	0	0	Severe	–	26	30
	Total	**0**	**0**	**0**	Total	**–**	**26**	**30**
Total number		**152**	**143**	**142**		**–**	**455**	**416**

*IA0* without initial assessment, *IA1* with initial assessment, without integration of results into electronic patient records, *IA2* with initial assessment, with integration of results into electronic patient records

*Since some symptoms appeared in both physician and nurse documentation, the total number of assessed symptoms does not equal the sum

The number of initiated supportive measures was lowest in the sample without initial assessment (IA0) followed by the sample with initial assessment (IA1) and highest in the sample where initial assessment results were represented in the clinical records (IA2). Results of the comparison of samples without and with an initial assessment regarding the number of initiated supportive measures are summarized in Table 2.

**Table 2:** Comparison of samples without/with initial assessment regarding the number of initiated supportive measures (n = 100/sample)

Symptom (day of admission)	Supportive measure (day of admission + day after)	Sample		
		IA0	IA1	IA2
Reduced emotional function	Non-pharmacological interventions against emotional burden (psycho-oncology, spiritual care, nursing consultation)	9	17	20
Fatigue	Blood transfusion (fatigue through anemia)	0	0	2
	Physiotherapy, sports and exercise therapy	6	17	25
Nausea/vomiting	Antiemetic medication	6	4	5
Pain	Pain medication	27	16	21
	Non-pharmacological interventions against pain (anesthesia council, other specialist councils, nursing pain management/consultation, mouth rinse)	6	4	6
Dyspnea	Medication against dyspnea	0	0	1
	Non-pharmacological interventions for ventilation/against dyspnea (inhalation, breath-stimulating rub, logopedics, O_2,_ breathing training)	5	6	10
Insomnia	Medication for sleep and sedation (including antidepressants for night)	9	9	9
Appetite loss	Medication against appetite loss	0	0	2
	Non-pharmacological interventions for nutritional promotion (nutrition specialist, logopedics)	19	25	26
Constipation	Laxatives	5	7	3
Diarrhea	Medication against diarrhea	1	0	0
Non-specific supportive measures	Occupational therapy	2	3	10
	Palliative council	2	3	1
	Oncologic care round	8	3	6
Total number		**105**	**114**	**147**

*IA0* without initial assessment, *IA1* with initial assessment, without integration of results into electronic patient records, *IA2* with initial assessment, with integration of results into electronic patient records

Regarding symptom monitoring, the three samples (SM0, SM1, SM2) had similar sociodemographic and clinical characteristics (Additional file 7). In comparison with the clinical records, the monitoring identified many additional symptoms. Results of the comparison of clinical records and the symptom monitoring regarding the number of identified symptoms are summarized in Table 3.

**Table 3:** Comparison of clinical records and symptom monitoring regarding the number of identified symptoms (n = 100/sample)

Symptom (random treatment day)	Clinical documentation*				Symptom monitoring (EORTC single items)			
	Sample	SM0	SM1	SM2	Sample	SM0	SM1	SM2
Depression	Physician	2	3	0	Moderate	–	41	21
	Nurse	0	2	1	Severe	–	15	26
	Total	**2**	**5**	**1**	Total	**–**	**56**	**47**
Skin problems	Physician	4	2	8	Moderate	–	16	14
	Nurse	0	3	3	Severe	–	13	18
	Total	**4**	**5**	**10**	Total	**–**	**29**	**32**
Nausea	Physician	4	6	5	Moderate	–	19	18
	Nurse	1	5	1	Severe	–	16	10
	Total	**4**	**10**	**6**	Total	**–**	**35**	**28**
Vomiting	Physician	0	0	0	Moderate	–	5	5
	Nurse	0	3	1	Severe	–	9	2
	Total	**0**	**3**	**1**	Total	**–**	**14**	**7**
Pain	Physician	8	18	24	Moderate	–	19	14
	Nurse	11	16	18	Severe	–	9	8
	Total	**14**	**27**	**34**	Total	**–**	**28**	**22**
Insomnia	Physician	1	3	5	Moderate	–	24	42
	Nurse	10	5	13	Severe	–	29	19
	Total	**11**	**8**	**17**	Total	**–**	**53**	**61**
Appetite loss	Physician	0	0	1	Moderate	–	24	26
	Nurse	0	0	2	Severe	–	18	15
	Total	**0**	**0**	**2**	Total	**–**	**42**	**41**
Constipation	Physician	3	8	8	Moderate	–	22	13
	Nurse	0	6	4	Severe	–	17	11
	Total	**3**	**12**	**10**	Total	**–**	**39**	**24**
Diarrhea	Physician	2	2	2	Moderate	–	4	13
	Nurse	1	1	3	Severe	–	2	5
	Total	**3**	**3**	**4**	Total	**–**	**6**	**18**
Tiredness	Physician	1	0	0	Moderate	–	41	41
	Nurse	0	0	0	Severe	–	33	34
	Total	**1**	**0**	**0**	Total	**–**	**74**	**75**
Weakness	Physician	2	0	1	Moderate	–	36	32
	Nurse	0	1	2	Severe	–	29	29
	Total	**2**	**1**	**3**	Total	**–**	**65**	**61**
Total number		**44**	**74**	**88**		**–**	**441**	**416**

*SM0* without symptom monitoring, *SM1* with symptom monitoring, without integration of results into electronic patient records, *SM2* with symptom monitoring, with integration of results into electronic patient records

*Since some symptoms appeared in both physician and nurse documentation, the total number of assessed symptoms does not equal the sum

The number of initiated supportive measures was lowest in the sample without symptom monitoring (SM0) followed by the sample with symptom monitoring (SM1) and highest in the sample where monitoring results were represented in the clinical records (SM2). Results of the comparison of samples without and with an initial assessment regarding the number of initiated supportive measures are summarized in Table 4.

**Table 4:** Comparison of samples without/with symptom monitoring regarding the number of initiated supportive measures (n = 100/sample)

Symptom (random treatment day)	Supportive measure (random treatment day + day after)	Sample		
		SM0	SM1	SM2
Depression	Non-pharmacological interventions against emotional burden (psycho-oncology, spiritual care, nursing consultation)	2	5	4
Skin problems	Non-pharmacological interventions against skin problems (skincare, nursing consultation, specialist councils)	1	1	5
Nausea, vomiting	Antiemetic medication	9	13	9
	Non-pharmacological interventions against nausea (nursing consultation)	1	0	0
Pain	Pain medication	17	25	25
	Non-pharmacological interventions against pain (anesthesia council, other specialist councils, nursing pain management/consultation, mouth rinse)	3	6	6
Insomnia	Medication for sleep and sedation (including antidepressants for night)	4	6	9
Appetite loss	Medication against appetite loss	0	0	1
	Non-pharmacological interventions for nutritional promotion (nutrition specialist, logopedics)	6	7	9
Constipation	Laxatives	5	12	11
	Non-pharmacological interventions against constipation (nursing consultation)	1	0	0
Diarrhea	Medication against diarrhea	3	1	0
Tiredness, weakness	Physiotherapy, sports and exercise therapy	6	6	7
Non-specific supportive measures	Occupational therapy	0	2	6
	Palliative council	1	0	2
	Oncologic care round	1	1	8
Total number		**60**	**85**	**102**

*SM0* without symptom monitoring, *SM1* with symptom monitoring, without integration of results into electronic patient records, *SM2* with symptom monitoring, with integration of results into electronic patient records

### Feasibility and acceptance

Not all patients could be approached. Barriers were time constraints of the patients (e.g. owing to scheduled treatments) resulting in a mismatch between patients’ availability and the availability of the research assistants. The COVID-19 pandemic resulting in the necessary adaptation of clinical routines caused further barriers.

If necessary (e.g. need for continuous support or reminder), some patients were approached repeatedly and the contacts documented accordingly. With respect to their autonomy and to reduce burden, patients who firmly stated their unwillingness to complete the e-PRO assessment at the first contact were not approached again.

Between 11/2019 and 02/2021, on 179 days a total of 1597 patient contacts were documented. Out of these contacts, n = 1355 (84.9%) resulted in the completion of one e-PRO assessment. Regarding the initial assessment, 436 contacts resulted in n = 355 (81.4%) completions; regarding symptom monitoring, 1117 contacts resulted in n = 967 (86.6%) completions; and regarding final assessment, which was introduced later in the process, 44 contacts resulted in n = 33 (75.0%) completions.

Depending on their actual health condition and their familiarity with electronic devices, patients could complete the e-PRO assessment on their own or with support from research assistants. Despite their ability to complete the assessment independently, many patients preferred the support (e.g. completing together with research assistants), appreciating the personal contact and communication. Regarding sex, age, and diagnosis of the patients, contacts resulting in non-completion were comparable to contacts resulting in completion (Additional file 8).

On average, more than one-third of the total number of all in-patients completed one e-PRO assessment every day. The main reasons for non-completion were health-related (e.g. high symptom burden, mild cognitive impairment) or lacking motivation and perception of benefit. Almost half of the reasons were related to specific conditions (e.g. forgetting to complete, other priorities) instead of a general refusal of the e-PRO assessment (Table 5).

**Table 5:** Reasons for non-completion of the e-PRO assessment (n = 255 reasons stated in n = 235 patient contacts)

General reasons (n = 129 reasons)	n (%)
Does not want to complete (no reason mentioned)	47 (18.4)
Completion too much burden (e. g. high symptom burden, poor general health status)	36 (14.1)
No motivation/does not see sense (e. g. no change in health status, no reaction on results anyway)	21 (8.2)
Mild cognitive impairment, problems regarding communication and/or understanding	19 (7.5)
General dissatisfaction (e. g. with treatment)	4 (1.6)
Does not like questions/answers	2 (0.8)
Specific reasons, but willing to complete in general (n = 126 reasons)	
Intention to complete independently later, but did not	52 (20.4)
Temporary (e. g. other priorities like family visit, high burden, upcoming therapy)	44 (17.3)
Forthcoming discharge/transfer	21 (8.2)
Technical problems	9 (3.5)

Barriers comprised technical and organizational issues e.g. Wi-Fi problems, interruption of the assessment completion (e.g. due to diagnostics, therapy), staff turnover as well as HCPs lack of time, doubts regarding the benefit of e-PROs, or a lack of use of the results by the majority of the clinical staff. The latter barriers were addressed during on-site and in team meetings supporting the implementation process.

### Required resources

For all 1354 patient contacts resulting in the completion of an e-PRO assessment, data regarding the duration were documented. On average 5.5 ± 5.3 (1–60) minutes per contact were needed to explain or support the completion of an e-PRO assessment.

On average, 11.4 ± SD 6.8 (1–60) minutes were required for supporting the initial assessment (354 analyzed patient contacts), 3.3 ± 2.0 (1–17) minutes for supporting symptom monitoring (967 analyzed patient contacts), and 6.2 ± 3.8 (1–15) minutes for supporting the final assessment (33 analyzed patient contacts).

## Discussion

This study focused on the implementation of e-PRO assessment into the routine practice of a radiation oncology clinic. So far, only a few pilot studies investigated a repeated assessment of e-PROs in an oncologic inpatient setting [39, 40]. Our study was conducted under “real-life” clinical conditions instead of study conditions to achieve results most representative for routine practice.

Comparison with clinical records indicated a large potential clinical benefit of e-PRO assessment regarding symptom identification. As in other studies [41], the assessments revealed an overall high symptom burden before and during treatment [5, 6]. While identifying many more issues compared to the documentation of clinical staff, e-PRO assessment seems to be most beneficial for detecting less “visible” symptoms such as fatigue or tiredness and often overlooked problems like financial difficulties, all of which can have a large impact on HRQOL [42, 43]. As in previous studies [5, 8], results also indicate the potential of e-PRO assessment to improve symptom management by leading to the initiation of more supportive measures. The integration into the electronic clinical records appears to further increase the awareness of the symptoms and the initiation of supportive measures.

The results provide comprehensive insight into the potential and requirements of e-PRO assessment complementing routine documentation. It became evident that the complex process of implementation requires flexibility, continuous optimization, and evaluation to adapt the procedure based on the knowledge gained throughout the process. In line with the EORTC manual on the use of EORTC measures in clinical practice [44], the importance of initial analysis of setting and current practice became evident for assessment of the general probability of a successful implementation. This includes also technical preconditions and integrability of the e-PRO assessment into clinical workflows.

Process evaluation indicated good feasibility, although the technical integration into the electronic patient records was challenging, a finding similar to other studies [45, 46]. As the support of the clinical IT turned out to be a crucial factor for successful integration, future implementations should consider an adequate amount of resources for IT personnel for a fast realization. Aiming to use existing ressources effectively, a multicenter implementation should start with a single center to realize the complex technical integration, gain experience, and make adaptations that can be used by the following centers. The basic e-PRO assessment implemented in inpatient radiation oncology will be easily adaptable for other inpatient clinics of the Krukenberg Cancer Centre considering setting-specific needs, patient characteristics and outcomes of relevance. An implementation into outpatient settings may require adapted approaches according to the different workflows. In a follow-up project for the outpatient radiation oncology clinic, e-PROs will be assessed via mobile tablets during the waiting times for therapy/consultation or from home via secure individualized access for patients with respective knowledge and technology.

Previous studies report cancer patients' willingness and ability to complete PROs regularly via electronic devices [5, 47] as relevant aspects for successful implementation [39, 40]. The results of this study confirm these findings and show an overall good acceptance. However, to maintain motivation, patient-reported issues must be addressed by HCPs and tailored measures initiated as patients have to recognize the consequences of their self-assessments [8, 29].

Instruments and procedures should also be acceptable for HCPs to facilitate the implementation into routine care [23, 24, 26]. To generate clinical benefit, the main challenge is the integration into the clinical workflow i.e. making “data actionable” [44]. In this respect, motivating HCPs to use the results in their routine practice seems to be most challenging. The main reason may be that changes in established clinical practice disrupt the existing "status quo", as they require questioning old beliefs and learning of new practices within existing contextual factors [48, 49]. Similar to other efforts to change established routines e.g. in quality improvement [50–52], the implementation and use of e-PRO assessment represents a multifaceted change process of existing systems and routines, influenced by the relationships between patients and clinical staff, their roles and structural conditions [53]. The experiences of our study suggest, that this complex process requires additional efforts concerning the motivation of all relevant stakeholders and the adjustment of overexpectations [54] as those changes need time.

Unlike other research from Germany [55], in our study HCPs only roughly self-estimated their familiarity with the concept of PROs as the survey was intended to gain basic knowledge regarding potential training needs. While a lack of basic knowledge regarding PROs may be a barrier for implementation, results suggest that even familiarity with the concept does not guarantee the use of PROs and their results in clinical practice. To enhance acceptance and motivation, we included HCPs in the planning process and decisions on instruments and clinical procedures [23], provided targeted training and recommendations for clinical use [56], and discussed relevant issues in team meetings [10]. However, these measures still appeared to be insufficient to ensure routine use of e-PRO assessments. As the results indicate improved symptom management already with few team members using the results of the e-PRO assessment, routine use by the whole multi-professional team may further increase the clinical benefit. Particularly motivated HCPs acting as “change champions” [46] could facilitate this development since their status as part of the clinical team may be more persuasive than external motivation. Furthermore, systematically developed targeted training for HCPs is needed regarding benefits of e-PRO assessment, PRO-based communication, PRO-based action with supportive measures, and self-management advice [26, 56–58]. Within this study, an e-learning course was developed but not yet used to educate HCPs. The future application may increase HCPs’ awareness and motivation to utilize the results of the e-PRO assessment.

To our knowledge, this is the first study evaluating the duration of patient contacts to explain and/or support the completion of e-PRO assessments, estimating necessary resources for routine implementation. Results indicate on average an additional workload of 5 to 6 min per patient to complete any e-PRO assessment, resulting in 150 to 180 additional minutes per day for 30 inpatients. As studies report that the integration of PROs did not increase the required time for consultations [47, 58], the explanation and/or support may be the most time-consuming aspect of the application of e-PRO assessment in clinical practice. However, due to our study design and limited resources of regular staff, this support was provided by the study staff. It might be expected that integration into the clinical routine of HCPs caring for the patients (e.g. reminding or supporting patients during ward rounds) would require less time. In addition, better symptom identification leading to more supportive measures, as indicated in this study, has the potential of reducing workload due to the prevention of adverse events, e.g. the risk of readmission [39]. Studies show that PROs are independent prognostic factors for important clinical outcomes such as survival [59, 60] and can therefore be used as decision aids when planning or adapting individual cancer therapy. The potential use of e-PRO assessment regarding the prevention of adverse events and optimization of therapy might be also beneficial for cost-efficacy in healthcare [61, 62].

Limitations of this implementation study are the single-center design and the inclusion of research assistants in the process. The majority of the patient contacts took place during the COVID-19 pandemic, resulting in extraordinary conditions e.g. lower number of admissions, only one patient per room, quarantined patients, different workflows/priorities for the clinical staff, and the need for interrupting the implementation process completely over several months. As described in another study [63], these pandemic conditions worked as a massive barrier by hindering and interrupting the implementation process, potentially reducing the acceptance of patients as well as staff and therefore preventing the e-PRO assessment to reach its full potential regarding potential benefit. Due to the anonymized documentation, contacts instead of persons were analyzed and the data have to be interpreted accordingly. A strength of this study is the combined evaluation of clinical benefit, feasibility/acceptance, and required resources of e-pro assessment based on a large number of analyzed patients and patient contacts, aiming to come as close to routine clinical conditions as possible. Our study did not aim for generalizability of the results but for a comprehensive evaluation of the complex and challenging implementation of e-PROs under routine conditionions to gain experience and knowledge for future implementations. With regard to setting-specific characteristics that may result in different symptoms and supportive measures of relevance, the methodologial approach, focusing on automated and criteria-based data extraction as well as proctocolled patient contacts, can be reproduced in other studies to systematically evaluate the implementation process.

Future research should focus on long-term implementations in different oncologic settings, apply a multi-center design starting with a pilot center and aim for routine clinical conditions to examine e-PRO assessment in “real-life” application contexts. Scientifically based implementation concepts [50–52] and models could be adapted and specified for this application to further enhance and facilitate the implementation of e-PRO assessment. Also, targeted educational interventions to use e-PRO-assessment [56], including communication strategies for the integration of e-PRO results into patient consultations [57] should be developed. From a technical perspective, the optimization of the user-friendliness of existing e-PRO assessment systems may facilitate their usability in the clinical routine.

## Conclusion

E-PRO assessment in an oncologic inpatient setting can support symptom identification and documentation by capturing additional self-reported burden of patients compared to the documentation of HCPs. Furthermore, it may lead to more supportive measures, especially if the results are easily visible due to their integration into the electronic patient records. While a repeated completion of e-PRO assessment appears to be acceptable for many patients, the challenge of making data “actionable” within the clinical workflow and motivating clinical staff became evident. The implementation of e-PRO assessment can be regarded as a complex process of permanent adaptation and optimization focusing on the integrability into existing technical systems and clinical workflows. In this context, the time needed to explain or support the completion of the assessment also has to be considered.

## Supplementary Information

**Additional file 1:** Overview of the process of PRO assessment from a technical viewpoint.

**Additional file 2:** Sociodemographic and clinical characteristics of patients completing the initial assessment with EORTC QLQ-C30 (n = 568), n (%) unless stated otherwise.

**Additional file 3:** Results of the initial assessment with EORTC QLQ-C30, scales 0-100, traffic light system based on Giesinger et al. [35] and Lehmann et al. [38] (n = 568 patients).

**Additional file 4:** Sociodemographic and clinical characteristics of patients completing the symptom monitoring with EORTC single items (n = 344), n (%) unless stated otherwise.

**Additional file 5:** Results of symptom monitoring with EORTC single items, scales 0-100, traffic light system based on tripartition (n = 1774 assessment times of n=344 patients, 1-36 times per patient).

**Additional file 6:** Sociodemographic and clinical characteristics of the three random samples (n=100 each) for the analysis of clinical records regarding the potential clinical benefit of the initial assessment.

**Additional file 7:** Sociodemographic and clinical characteristics of the three random samples (n = 100 each) for the analysis of clinical records regarding the potential clinical benefit of the symptom monitoring.

**Additional file 8:** Sociodemographic and clinical characteristics of patient contacts resulting in completion/non-completion of the e-PRO assessment, n (%)*.

## Data Availability

The scope of this implementation study was to improve the quality of care in clinical routine through the implementation of e-PRO assessment focusing on the evaluation of the process under “real-life” conditions and not under clinical trial conditions. In accordance with the ethics committee, no informed consent of patients completing the e-PRO assessment was obtained. Therefore, due to data protection and ethical regulations, the anonymized datasets generated or analyzed during the study cannot be provided.

## Abbreviations

CHES: Computer-based Health Evaluation System
e-PRO(s): Electronic patient reported outcome(s)
EORTC: European Organisation of Research and Treatment of Cancer
HCP(s): Healthcare professional(s)
HRQOL: Health-related quality of life
MRC: Medical Research Council
PRO(s): Patient-reported outcome(s)
