# Supplementary figures and images for: An implementation study of electronic assessment of patient-reported outcomes in inpatient radiation oncology
Source: J Patient Rep Outcomes. 2022 Jul 19;6:77. doi: 10.1186/s41687-022-00478-3 (PMC9296709; doi:10.1186/s41687-022-00478-3)

Overview of the process of PRO assessment from a technical viewpoint

**
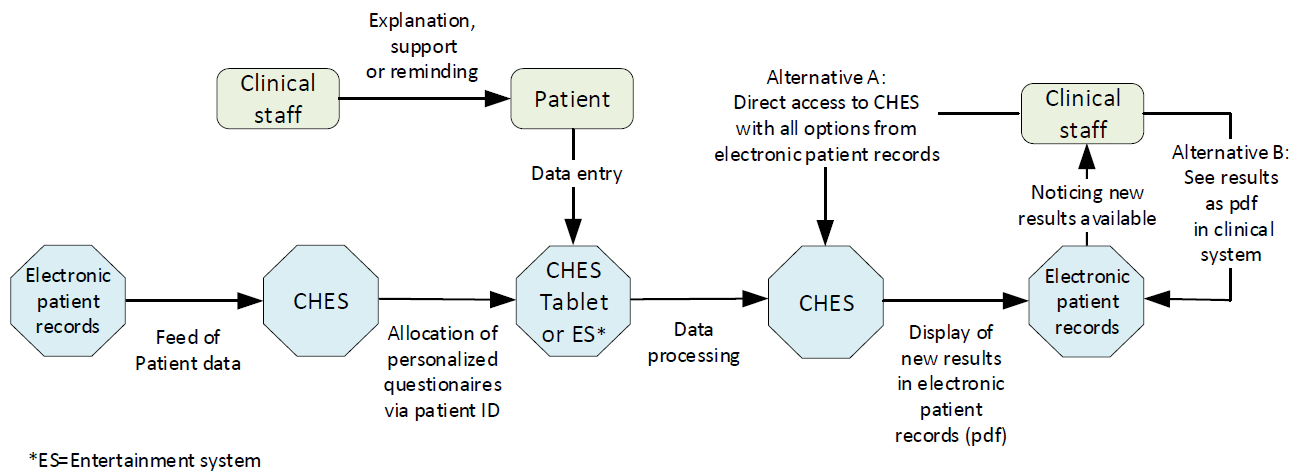
**

Supplement: Supplementary file 1 — Additional file 1: Overview of the process of PRO assessment from a technical viewpoint. [file 41687_2022_478_MOESM1_ESM.docx]
