# Supplementary material for: An implementation study of electronic assessment of patient-reported outcomes in inpatient radiation oncology
Source: J Patient Rep Outcomes. 2022 Jul 19;6:77. doi: 10.1186/s41687-022-00478-3 (PMC9296709; doi:10.1186/s41687-022-00478-3)
Supplement: Supplementary file 2 — Additional file 2: Sociodemographic and clinical characteristics of patients completing the initial assessment with EORTC QLQ-C30 (n = 568), n (%) unless stated otherwise. [file 41687_2022_478_MOESM2_ESM.docx]

Sociodemographic and clinical characteristics of patients completing the initial assessment with EORTC QLQ-C30 (n=568), n (%) unless stated otherwise

| Sex |  |
| --- | --- |
| Male | 367 (64.6) |
| Female | 201 (35.4) |
| Age*, years ± SD | 64.9 ± 11.6 |
| < 50 | 42 (7.4) |
| 50-70 | 346 (60.9) |
| > 70 | 180 (31.7) |
| Primary tumor |  |
| Head/neck | 179 (31.5) |
| Lung | 167 (29.4) |
| Colorectal | 66 (11.6) |
| Gynaecological | 40 (7.0) |
| Upper GI tract | 35 (6.1) |
| Skin | 17 (3.0) |
| Kidney/urinary tract | 17 (3.0) |
| Brain | 14 (2.5) |
| Breast | 10 (1.8) |
| Other | 53 (9.3) |
| More than one primary tumor | 32 (5.6) |
| At least one secondary site | 391 (68.8) |

*Age based on the range between the date of birth and date of assessment.
