# Supplementary material for: An implementation study of electronic assessment of patient-reported outcomes in inpatient radiation oncology
Source: J Patient Rep Outcomes. 2022 Jul 19;6:77. doi: 10.1186/s41687-022-00478-3 (PMC9296709; doi:10.1186/s41687-022-00478-3)
Supplement: Supplementary file 3 — Additional file 3: Results of the initial assessment with EORTC QLQ-C30, scales 0-100, traffic light system based on Giesinger et al. [35] and Lehmann et al. [38] (n = 568 patients). [file 41687_2022_478_MOESM3_ESM.docx]

Results of the initial assessment with EORTC QLQ-C30, scales 0-100, traffic light system based on Giesinger et al. [35] and Lehmann et al. [38] (n=568 patients)

**
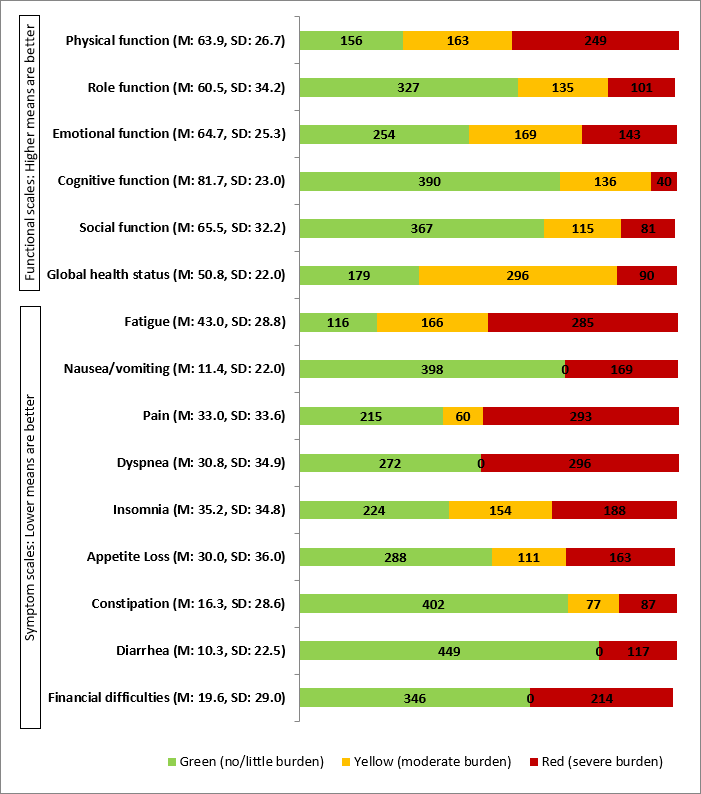
**
