# Supplementary material for: An implementation study of electronic assessment of patient-reported outcomes in inpatient radiation oncology
Source: J Patient Rep Outcomes. 2022 Jul 19;6:77. doi: 10.1186/s41687-022-00478-3 (PMC9296709; doi:10.1186/s41687-022-00478-3)
Supplement: Supplementary file 4 — Additional file 4: Sociodemographic and clinical characteristics of patients completing the symptom monitoring with EORTC single items (n = 344), n (%) unless stated otherwise. [file 41687_2022_478_MOESM4_ESM.docx]

Sociodemographic and clinical characteristics of patients completing the symptom monitoring with EORTC single items (n=344), n (%) unless stated otherwise

| Sex |  |
| --- | --- |
| Male | 216 (62.8) |
| Female | 128 (37.2) |
| Age*, years ± SD | 63.7 ± 11.0 |
| < 50 | 26 (7.6) |
| 50-70 | 230 (66.9) |
| > 70 | 88 (25.6) |
| Primary tumor |  |
| Head/neck | 113 (32.9) |
| Lung | 106 (30.8) |
| Colorectal | 32 (9.3) |
| Female genitals | 26 (7.6) |
| Upper GI tract | 23 (6.7) |
| Skin | 6 (1.7) |
| Kidney/urinary tract | 8 (2.3) |
| Brain | 9 (2.6) |
| Breast | 6 (1.7) |
| Other | 25 (7.3) |
| More than one primary tumor | 12 (3.5) |
| At least one secondary site | 234 (68.0) |

*Age based on the range between the date of birth and date of the first monitoring.
