# Supplementary material for: An implementation study of electronic assessment of patient-reported outcomes in inpatient radiation oncology
Source: J Patient Rep Outcomes. 2022 Jul 19;6:77. doi: 10.1186/s41687-022-00478-3 (PMC9296709; doi:10.1186/s41687-022-00478-3)
Supplement: Supplementary file 5 — Additional file 5: Results of symptom monitoring with EORTC single items, scales 0-100, traffic light system based on tripartition (n = 1774 assessment times of n=344 patients, 1-36 times per patient). [file 41687_2022_478_MOESM5_ESM.docx]

Results of symptom monitoring with EORTC single items, scales 0-100, traffic light system based on tripartition (n=1774 assessment times of n=344 patients, 1-36 times per patient)

**
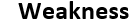

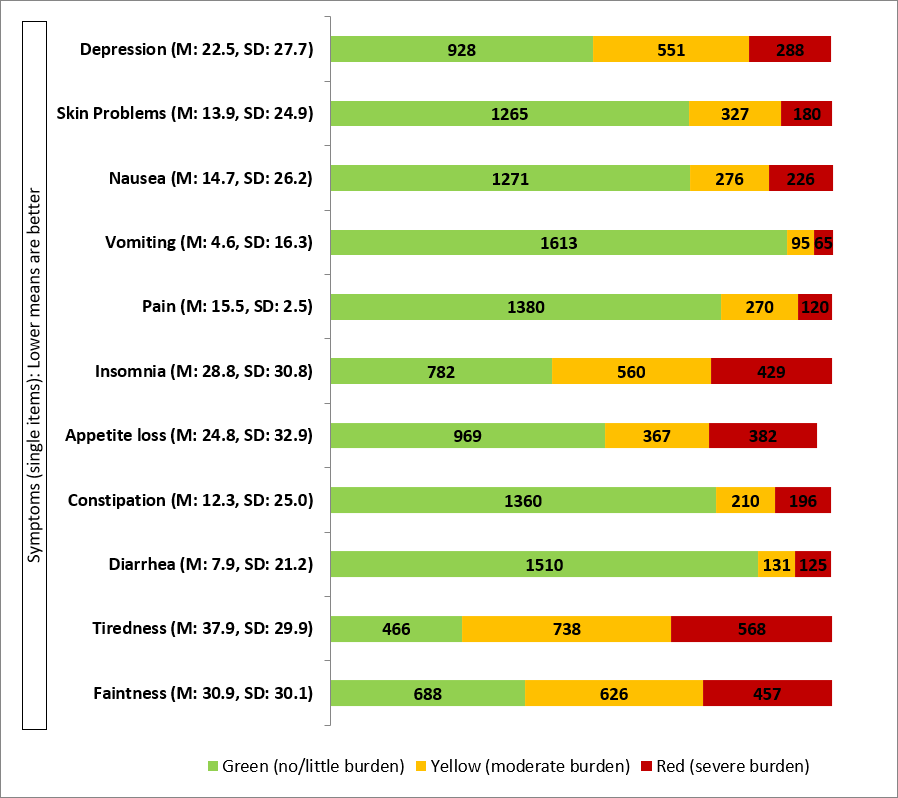
**
