# Supplementary material for: An implementation study of electronic assessment of patient-reported outcomes in inpatient radiation oncology
Source: J Patient Rep Outcomes. 2022 Jul 19;6:77. doi: 10.1186/s41687-022-00478-3 (PMC9296709; doi:10.1186/s41687-022-00478-3)
Supplement: Supplementary file 6 — Additional file 6: Sociodemographic and clinical characteristics of the three random samples (n=100 each) for the analysis of clinical records regarding the potential clinical benefit of the initial assessment. [file 41687_2022_478_MOESM6_ESM.docx]

Sociodemographic and clinical characteristics of the three random samples (n=100 each) for the analysis of clinical records regarding the potential clinical benefit of the initial assessment

| **Sample** | | **IA0** | **IA1** | **IA2** |
| --- | --- | --- | --- | --- |
| **Sex** | | | | |
| Male | 66 | | 56 | 67 |
| Female | 34 | | 44 | 33 |
| **Age (years)** | | | | |
| Mean | 65.3 | | 66.5 | 64.5 |
| SD | 10.8 | | 12.1 | 10.5 |
| Min  Max | 43.0  89.0 | | 41.0  94.0 | 38.0  89.0 |
| **Diagnosis** | | | | |
| Head/neck | 19 | | 28 | 32 |
| Lung | 40 | | 39 | 31 |
| Colorectal | 11 | | 7 | 13 |
| Female genitals | 5 | | 4 | 6 |
| Upper GI tract | 4 | | 7 | 4 |
| Skin | 2 | | 5 | 1 |
| Kidney/urinary tract | 3 | | 3 | 1 |
| Brain | 1 | | 6 | 3 |
| Breast | 3 | | 4 | 2 |
| Other | 13 | | 6 | 9 |
| More than one primary tumor | 1 | | 9 | 2 |
| At least one secondary site | 74 | | 77 | 65 |

**IA0:** Without initial assessment; **IA1:** Initial assessment without integration of results into clinical records;

**IA2:** Initial assessment with integration of results into clinical records.
