# Supplementary material for: An implementation study of electronic assessment of patient-reported outcomes in inpatient radiation oncology
Source: J Patient Rep Outcomes. 2022 Jul 19;6:77. doi: 10.1186/s41687-022-00478-3 (PMC9296709; doi:10.1186/s41687-022-00478-3)
Supplement: Supplementary file 7 — Additional file 7: Sociodemographic and clinical characteristics of the three random samples (n = 100 each) for the analysis of clinical records regarding the potential clinical benefit of the symptom monitoring. [file 41687_2022_478_MOESM7_ESM.docx]

Sociodemographic and clinical characteristics of the three random samples (n=100 each) for the analysis of clinical records regarding the potential clinical benefit of the symptom monitoring

| **Sample** | | **SM0** | **SM1** | **SM2** |
| --- | --- | --- | --- | --- |
| **Sex** | | | | |
| Male | 63 | | 62 | 65 |
| Female | 37 | | 38 | 35 |
| **Age (years)** | | | | |
| Mean | 64.3 | | 62.9 | 63.6 |
| SD | 12.1 | | 11.0 | 10.0 |
| Min  Max | 30.0  87.0 | | 31.0  90.0 | 34.0  87.0 |
| **Diagnosis** | | | | |
| Head/neck | 23 | | 31 | 32 |
| Lung | 30 | | 36 | 30 |
| Colorectal | 11 | | 5 | 11 |
| Female genitals | 9 | | 6 | 6 |
| Upper GI tract | 7 | | 13 | 4 |
| Skin | 2 | | 2 | 0 |
| Kidney/urinary tract | 2 | | 3 | 2 |
| Brain | 1 | | 3 | 4 |
| Breast | 1 | | 2 | 3 |
| Other | 14 | | 2 | 11 |
| More than one primary tumor | 0 | | 3 | 3 |
| At least one secondary site | 69 | | 70 | 76 |

**SM0:** Without symptom monitoring; **SM1:** With symptom monitoring, without integration of results into clinical records;

**SM2**: Symptom monitoring with integration of results into clinical records.
