# Supplementary material for: An implementation study of electronic assessment of patient-reported outcomes in inpatient radiation oncology
Source: J Patient Rep Outcomes. 2022 Jul 19;6:77. doi: 10.1186/s41687-022-00478-3 (PMC9296709; doi:10.1186/s41687-022-00478-3)
Supplement: Supplementary file 8 — Additional file 8: Sociodemographic and clinical characteristics of patient contacts resulting in completion/non-completion of the e-PRO assessment, n (%)*. [file 41687_2022_478_MOESM8_ESM.docx]

Sociodemographic and clinical characteristics of patient contacts resulting in completion/non-completion of the e-PRO assessment, n (%)*

| **Sample characteristics** | **Contacts resulting in**  **completion (n=1355)** | **Contacts resulting in non-completion (n=242)** |
| --- | --- | --- |
| **Sex** |  |  |
| Male | 831 (61.3) | 154 (63.6) |
| Female | 518 (38.2) | 83 (34.3) |
| **Age, years ± SD (range)** | 66.7 ± 10.9 (31-91) | 64.1 ± 11.2 (34-91) |
| **Age groups** |  |  |
| < 50 years | 53 (3.9) | 20 (8.3) |
| 50 - 70 years | 814 (60.1) | 156 (64.5) |
| > 70 years | 478 (35.3) | 61 (25.2) |
| **Diagnosis** |  |  |
| Head/neck | 435 (32.1) | 94 (38.8) |
| Lung | 363 (26.8) | 60 (24.8) |
| Colorectal | 95 (7.0) | 14 (5.8)) |
| Gynaecological | 117 (8.6) | 20 (8.3) |
| Upper GI tract | 67 (4.9) | 6 (2.5) |
| Skin | 38 (2.8) | 6 (2.5) |
| Kidney/urinary tract | 20 (1.5) | 6 (2.5) |
| Brain | 55 (4.1) | 7 (2.9) |
| Breast | 8 (0.6) | 0 (0.0) |
| Other | 157 (11.6) | 29 (12.0) |

*unless stated otherwise
